# 547. Risk Factors Associated with 30-Day Mortality in a Large Cohort of Patients who Received Remdesivir and Corticosteroids for Severe COVID-19

**DOI:** 10.1093/ofid/ofab466.746

**Published:** 2021-12-04

**Authors:** Kartik Gupta, Lea Monday, Milan Kaushik, George J Alangaden, Indira Brar

**Affiliations:** 1 Henry Ford Health System, Detroit, Michigan; 2 Wayne State University School of Medicine, Detroit, Michigan; 3 Henry Ford Hospital, Detroit, Michigan

## Abstract

**Background:**

Remdesivir (RDV), an antiviral agent, is approved by Food and Drug Administration (FDA) for the treatment of patients (pts) admitted with SARS-COV-2 infection (COVID-19). Earlier RDV studies (such as ACCT-1) prior to widespread use of corticosteroids (CS), showed a 30-day mortality of 11%. Advanced age, obesity, and certain comorbidities are known risk factors for death in COVID-19, but whether these risks vary in pts treated with RDV and CS is unknown. As of March 20, 2020 CS were routinely used for the treatment of pts admitted with COVID19 in our health care system. The objective of this study was to identify risk factors associated with 30 -Day mortality in a cohort of pts admitted with COVID-19 and who received RDV and CS.

**Methods:**

This retrospective cohort study evaluated pts admitted to a health system in South East Michigan with COVID-19 between March and November 2020 who received ≥1 dose RDV. Demographics, comorbidities, and characteristics including quick sequential organ failure assessment (qSOFA) score were collected and compared between patients who died versus survived. Primary outcome was 30 day mortality. Secondary outcomes were risk factors for death using logistic regression and time-to-event analysis.

**Results:**

A total of 1,591 pts received RDV and were included in the study; median age 67 years, 56% male and 18% Black. RDV use increased after emergency use authorization and FDA approval (Fig 1). Death within 30 days occurred in 15.3%. Patients who died were older males with higher rates of hypertension, kidney disease, diabetes, and were more likely to have qSOFA ≥2 on arrival (Table 1). In a multivariable logistic model, advanced age, male gender, pulmonary disease, CKD, obesity, and qSOFA≥2 were independent predictors of death (Figure 2). Among these, age and qSOFA≥2 were the most important risk factors (Figure 2).

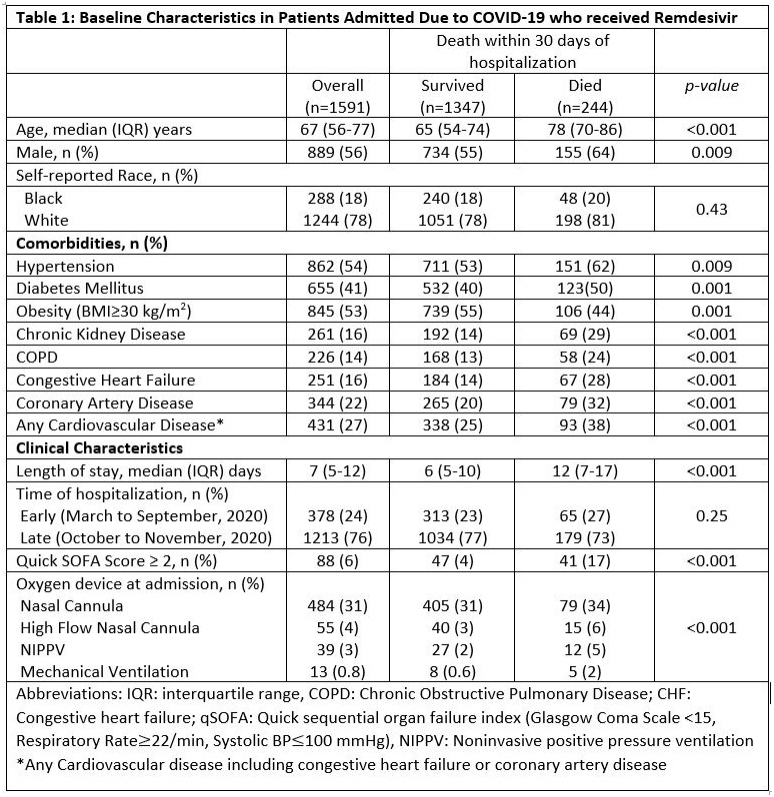

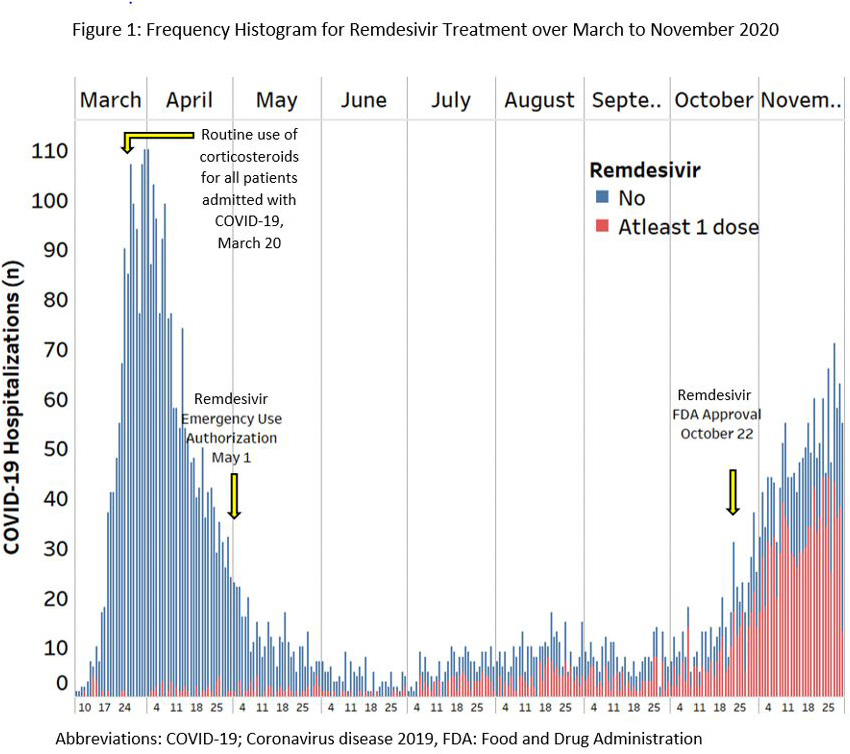

Patients receiving remdesivir (red) were included in the study. Routine use of corticosteroids was adopted on all patients in our health system beginning March 20, 2020. System-wide use of remdesivir increased following Food and Drug Administration approval in fall 2020.

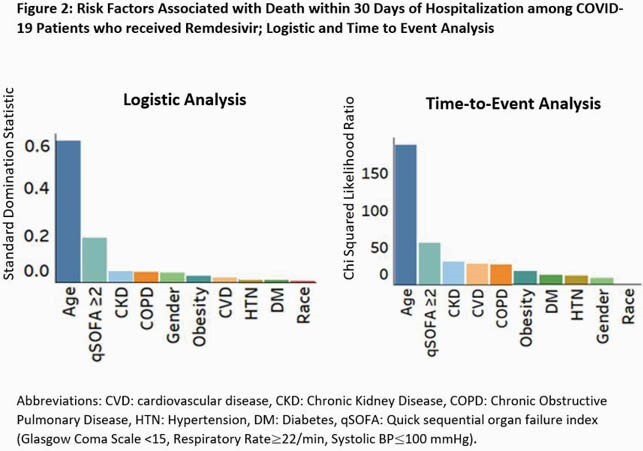

On both logistic regression and time-to-event analysis, advanced age and qSOFA ≥ 2 had the highest predictive value for mortality. Others comorbidities were similar and comparable in importance.

**Conclusion:**

The population in our Real-world study was older with more comorbidities as compared to ACCT-1, and the 30 day mortality was 15%. Despite the use of CS and RDV advanced age and qSOFA were the most important drivers of mortality. Future, therapeutic strategies need to focus on this group which is at the highest risk of dying from COVID-19 infection.

**Disclosures:**

**All Authors**: No reported disclosures

